# Aflatoxin contamination of maize flour in Kenya: Results from multi-city, multi-round surveillance

**DOI:** 10.1371/journal.pone.0336687

**Published:** 2025-11-13

**Authors:** Vivian Hoffmann, Boaz Ndisio, Allan Barasa, Sheila Okoth, Mike Murphy

**Affiliations:** 1 International Food Policy Research Institute, Washington, DC, United States of America; 2 Department of Economics, Carleton University, Ottawa, Canada; 3 Asperlabs Limited, Aflatoxin Management Services, Nairobi, Kenya; 4 Food Safety Division, Ministry of Health, Nairobi, Kenya; 5 Department of Biology, Faculty of Science and Technology, University of Nairobi, Nairobi, Kenya; University of Johannesburg, SOUTH AFRICA

## Abstract

Foodborne illness is a major source of the global burden of disease, but public monitoring of hazards in food systems is overwhelmingly focused on the formal sector in high income countries. We contribute to the development of an evidence base on food safety risk in low-income and informal settings by monitoring aflatoxin prevalence in maize flour in Kenya. Aflatoxin is a contaminant which causes liver cancer and has been linked to childhood stunting. We carry out systematic monitoring of formally and informally processed maize flour from a range of retail vendors across ten urban sites in Kenya and analyze aflatoxin levels in commercial samples. Samples were obtained every two months from February-December 2021 and 1255 samples in total were analyzed. Almost all samples (97%) showed detectable levels of aflatoxin, with 16% of tested samples exceeding the national regulatory limit of 10 ppb. Mean contamination levels are significantly higher (p < 0.001) in informal market samples (9.9 ppb) than in packaged flour in the formal sector (4.9 ppb). We find important seasonal variation in aflatoxin levels, which are highest in our June sample and lowest in December, which we attribute to variation in sourcing of maize grain. Our results demonstrate the need for policy interventions to reduce aflatoxin exposure in Kenya and demonstrate the utility of coordinated monitoring efforts to track levels of food safety risk in low-income settings.

## Introduction

The modernization of food systems toward larger-scale processing and traceable supply chains is expected to facilitate surveillance for foodborne hazards and compliance with food safety regulations [1]. However, comparative analysis of food safety in traditional versus modern sub-sectors is scarce, due to the fact that public surveillance systems typically omit informally traded foods. This omission implies that the evidence required for the identification of high-risk foods, and ultimately for the implementation of a risk-based approach to management of food safety, is lacking in many contexts.

Numerous studies have documented high levels of aflatoxin in maize grown and marketed in Kenya [2]. The contaminant is estimated to cause up to 26% of the global burden of the main type of liver cancer [3] and is implicated as a contributor to child stunting [4,5]. While reducing exposure to aflatoxin is a food safety priority of the Government of Kenya, systematic data on contamination is unavailable.

The objective of our study is to compare levels of aflatoxin contamination in maize flour available for sale to consumers by production process, location, and season in Kenya. To achieve this, we designed a systematic sampling plan for the surveillance of aflatoxin levels in maize flour sold packaged by registered firms and flour processed informally by “posho” mills. We collected six rounds of samples from multiple retailers over a one-year period in ten major metropolitan areas of Kenya. The resulting data fill a crucial gap in evidence required for risk-based management of aflatoxin contamination in Kenya, and allow us to test the hypothesis of whether compliance with the aflatoxin food safety regulation differs across the formal versus informal sector.

Several previous studies have analyzed marketed products for aflatoxin contamination. Due to its high levels of aflatoxin contamination, many of these studies have been conducted in Kenya. Lewis et al. compare contamination in marketed maize across districts in the wake of an aflatoxicosis epidemic and conclude that the sale of contaminated locally grown maize into markets constituted an ongoing source of contamination [6]. Daniel et al. find that homegrown maize in a high aflatoxin risk region is consistently more contaminated than purchased maize [7], while Hoffmann et al. report slightly lower contamination in homegrown maize based on samples collected from hammer mills over a broader geographical area [8]. Mutiga et al., using the same sample as Hoffmann et al., as well as a similar sample from a different part of the country, characterize the aflatoxin prevalence by agroecological zone in two regions of Kenya during separate years [9,10].

Three features distinguish the present paper from previous work. First, rather than selecting sampling sites based on agroecological conditions, as done by the above-referenced studies led by Mutiga, as well as others [11,12], we take as our sampling frame the maize available for purchase in geographically diverse urban areas across Kenya. Second, our analysis is based on six distinct data collection rounds, spaced throughout one calendar year. This allows us to characterize seasonal patterns of contamination in marketed grain, which may be influenced by the time since maize was harvested, and the region from which it was sourced. Finally, our sample includes formally milled, sifted, and packaged maize flour in addition to informally ground whole meal flour, enabling comparisons of food safety across the informal and formal sectors. Comparing contamination across sector (equivalently whole meal versus refined product type, as formally milled flour is almost always more refined than informally milled flour) is the most policy-relevant contribution of this work, as it can be used to provide actionable food safety information to consumers. Assessing the robustness of such a comparison across time and location is critical, as the relative riskiness of alternative food products may vary over the course of the year and across space.

## Methods

### Ethical review

A full research protocol was submitted to the International Food Policy Research Institute (IFPRI) Institutional Review Board and approved for exemption as data collection did not include information from human or animal subjects. All flour samples were purchased at market rates from public retail sites, so no additional permissions were required for field site access.

### Sampling strategy

The sampling strategy was designed to capture variation in aflatoxin contamination across product category, time, and location in a cost-effective manner. Both formally milled and branded maize flour and maize milled in small-scale hammer mills (posho) were included. Posho mill samples include maize flour milled from grain provided by the miller, and flour milled from customers’ grain (either purchased by the customer or home-grown). Samples were collected once every two months, over the course of a year, in ten urban centers. These urban centers were included in the sample based on their proximity to at least one industrial maize mill, and to achieve representation of maize-growing regions and international maize imports, specifically the regional trade hub of Mombasa and the Kajiado area which has a major road link to Tanzania, Kenya’s largest source of imported maize at the time [13]. While the urban focus of the sampling strategy implies some limitations, inclusion of rural sampling sites was not feasible within the budget available for this work.

**Fig 1:**
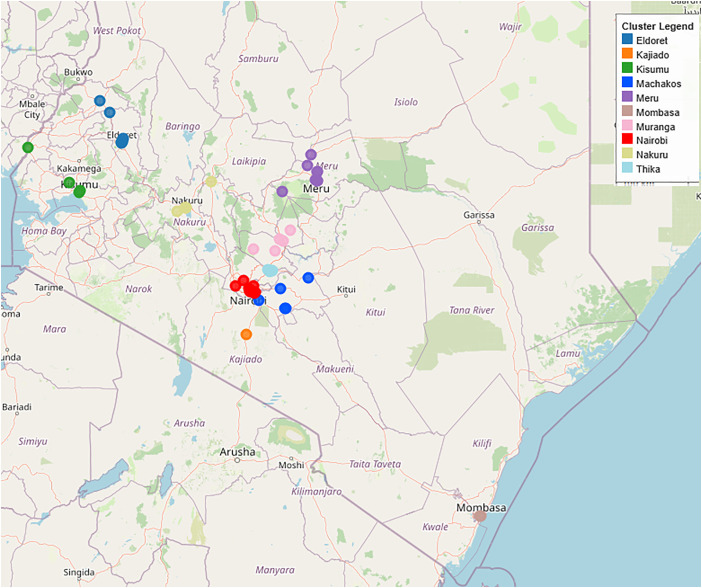
Map showing location of commercial mills, with points colored by sampling cluster. (https://www.openstreetmap.org).

To identify flour brands for sampling, we obtained a list of all grain milling firms formally registered with the Kenyan Bureau of Standards, and the maize flour brands associated with each of these. We aimed to link each brand to the maize-producing region or international port of entry from which at least some of the maize used to produce it was sourced, while acknowledging that maize is often transported across Kenya, with the pattern of this trade varying seasonally [14]. We used publicly available address data and Google Maps software to determine the GPS location associated with the identified firms. Each of the 64 firms for which geographic data were found were then assigned to one of 10 clusters associated with a metropolitan area based on geographic proximity (Fig 1). These metropolitan areas constituted the sampling sites for maize flour collection: Eldoret, Kajiado, Kisumu, Machakos, Meru, Mombasa, Nairobi, Nakuru, Nyeri, and Thika. Firms with mills in multiple locations were mapped to multiple clusters, and samples associated with these mills were mapped to mill nearest to their location of purchase.

At each site, a listing of brands was conducted by an enumerator recruited from within or near that site. Enumerators visited a total of 363 retailers across sites, including supermarkets and smaller shops selling maize flour, and recorded the brands available for sale at each of these. These visits were carried out in December 2020 and were used to build a list of formal maize flour brands available at each of the ten sampling sites. For firms offering multiple brands, the lowest-priced brand was retained in the sample, as higher-priced brands tend to be more refined and thus at lower risk of aflatoxin contamination. Blended flour products containing ingredients other than maize were likewise dropped.

We then reviewed the availability of each brand to distinguish between brands sold nationally and regional brands. We defined a brand as nationally distributed if it was sold in at least half of the ten sampling sites; six brands met this threshold. We refer to these six as “national brands” in the analysis, and all other brands as “regional brands” (regional brands were not necessarily limited to one location, with some found at up to three sites). Samples of national brands were collected from five randomly selected locations at which they had been observed during listing, with one sample targeted per location.

Samples of regional brands could be collected from up to three locations: the nearest to the location of the mill producing that brand, and up to two additional locations where the brand was observed during the listing exercise. The target number of samples of regional brands per site was 1 if the brand was available in at least three locations (for a total of 3 samples), 2 if the brand was linked to two locations (4 samples) or 3 if the brand was linked to only one location (3 samples). This strategy aimed to achieve variation in sampling sites within-brand to the extent feasible, in order to avoid collecting repeated samples from a single manufacturing lot (which is not typically printed on flour packaging). If multiple samples of a brand were collected from a single site, enumerators were instructed to obtain these from different shops.

### Maize flour collection

Every two months, enumerators visited retail locations, markets, and small-scale hammer mills (posho mills) in each of the 10 sampling sites. We refer to formally packaged samples as “branded” samples to distinguish them from samples purchased at posho mills. Posho mill samples include both maize flour milled from grain provided by the miller, and flour milled from customers’ grain (either purchased by the customer or home-grown). Table 1 shows the schedule of maize flour collection visits completed in 2021.

**Table 1:** Calendar of data collection visits for aflatoxin monitoring in ten urban areas.

Round	Months
1	February-March
2	April
3	June
4	August
5	October
6	December

Each round, enumerators were provided with a list of the maize flour brands for which they were to obtain samples, and the number of samples targeted for each brand. At each of five posho mills per sampling site, a 1 kg sample of flour from the miller’s supply was obtained if available, or from purchased grain brought in by a customer if the miller did not have flour available at the time of the visit. Additionally, up to five samples of flour processed from homegrown maize were obtained from mill customers, if available. These samples could be collected at the same mill, though each sample came from a different customer. This resulted in a total of up to 10 posho samples per enumerator per round. Customers were asked for only 200 g samples of their home-grown grain, based on the observation that customers typically brought small amounts of maize for milling, and were often not willing to part with grain they had grown even for above-market rate compensation.

For each branded and posho flour sample, the enumerator purchased either a 1 kg (if available) or 2 kg bag and completed an electronic form on a tablet computer, through which data on the price, origin, and brand ID were recorded. Unique identifiers generated by the data collection software were recorded on flour samples, and these were sent to a laboratory at the University of Nairobi via courier within 48 hours of collection.

### Laboratory analysis

An extraction solution of 70% of methanol was prepared using 30 mL of distilled water to 70 ml of methanol (reagent grade) for each sample to be tested. Each sample of maize flour was evenly mixed, 20 grams of the sample were weighed out, and 100 ml of the extraction solution was added and mixed in a shaker for 10 minutes. Particulate matter was allowed to settle and 5–10 ml of the extract was filtered through a Whatman #1 filter paper. Analysis of total aflatoxins was then conducted on the extract using the Helica Biosystems Inc Total Aflatoxin ELISA Low Matrix Kit according to manufacturer’s instructions (Helica-Hygiena, Hygiena International Ltd, 26 Frederick Sanger Road, Guilford, GU2 7YD, United Kingdom). The upper and lower limits of detection were 0.5 ppb and 20 ppb respectively. The limit of quantification was 1.0ppb. The recovery percentage for this method is 85% in 80% acetonitrile.

## Results

### Coverage

**Fig 2:**
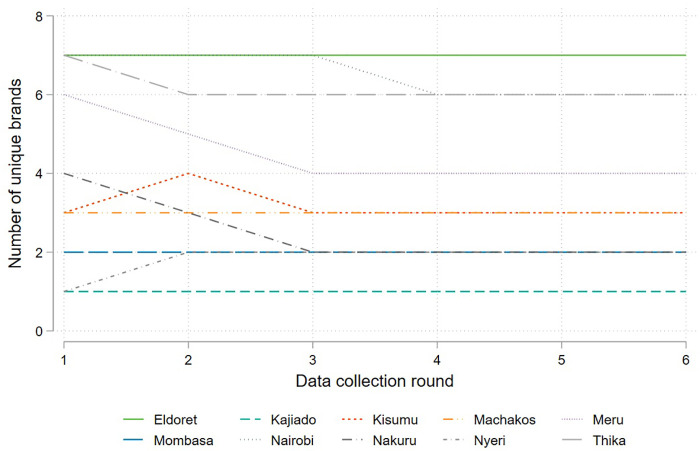
Number of samples of unique packaged maize flour brands collected, by round and mill location.

The sample of brands identified through the initial listing consisted of 63 unique brands (including national brands). Of these, only 48 brands were found during the first round of data collection. Discussions with a milling industry association representative suggested that this was due to a greater variety of brands being offered around the Christmas season when the brand listing was conducted, as demand is highest at this time of year. Enumerators continued to check for the availability of these brands in subsequent data collection rounds. The total number of brands for which samples were obtained per milling location was therefore relatively stable across data collection rounds, as shown in Fig 2 below.

Table 2 summarizes the total number of maize samples collected and analyzed by the milling location to which the sample was linked. In total, 1377 samples were purchased. Of these, 122 were either delayed in transit or suffered damage to the sample or the identifying label, resulting in a total of 1255 samples (91.1% of those purchased) which were successfully processed and analyzed. These comprised 695 branded flour samples and 560 samples from posho mills.

**Table 2:** Total maize flour samples analyzed for aflatoxin contamination across all rounds, disaggregated by flour source.

	Packaged			Posho		
	All	*National*	*Regional*	All	*Purchased grains*	*Homegrown grains*
**Eldoret**	**95**	*0*	*95*	**60**	*55*	*5*
**Kajiado**	**17**	*0*	*17*	**57**	*30*	*27*
**Kisumu**	**47**	*0*	*47*	**53**	*41*	*12*
**Machakos**	**48**	*0*	*48*	**58**	*48*	*10*
**Meru**	**66**	*0*	*66*	**57**	*53*	*4*
**Mombasa**	**53**	*30*	*23*	**59**	*54*	*5*
**Nairobi**	**138**	*30*	*108*	**48**	*48*	*0*
**Nakuru**	**45**	*27*	*18*	**60**	*55*	*5*
**Nyeri**	**33**	*0*	*33*	**55**	*50*	*5*
**Thika**	**153**	*87*	*66*	**53**	*49*	*4*
**Total**	**695**	*174*	*521*	**560**	*483*	*77*

### Contamination levels

**Fig 3:**
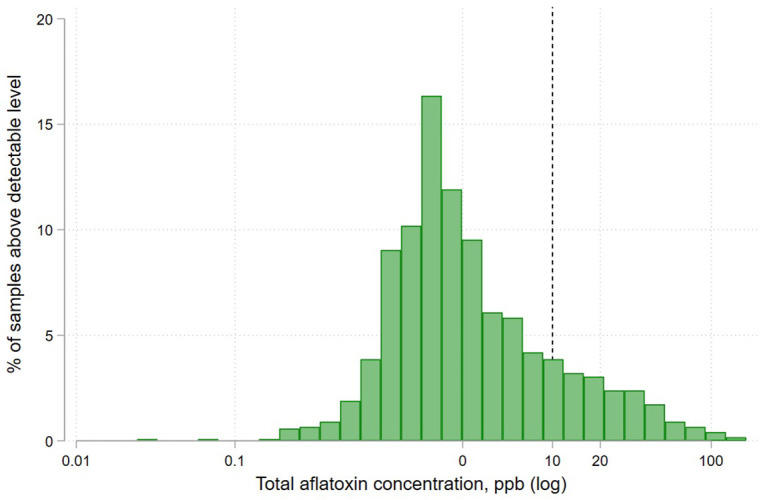
Distribution of total aflatoxin contamination levels in maize flour samples. *Note:* samples with aflatoxin below the lower limit of detection are excluded from this figure.

Of the 1,255 samples analyzed, 97% showed a detectable level of aflatoxin, with 16% of all samples testing above the regulatory limit of 10 ppb. Fig 3 shows the distribution of contamination levels for samples above the detectable level of aflatoxin.

**Fig 4:**
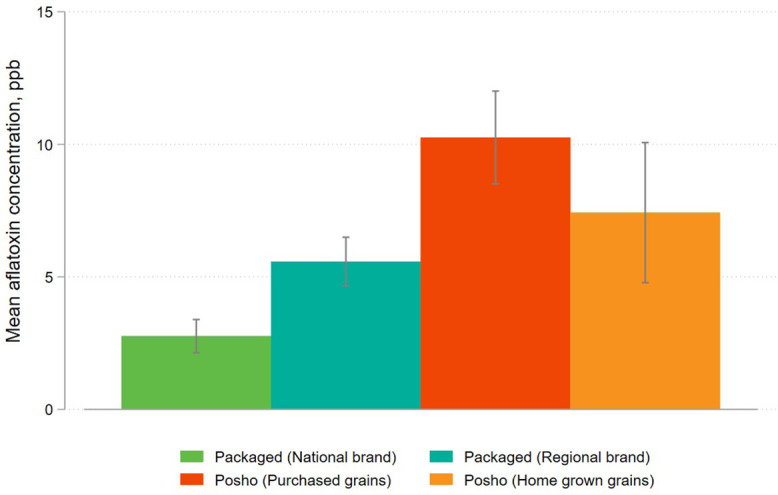
Mean aflatoxin contamination levels in maize flour samples, disaggregated by flour source.

Fig 4 shows contamination levels by flour type, pooling all rounds of data collection and sampling sites. Contamination levels varied significantly between branded flour and informally milled posho flour. Pooling across brand types and sources, branded flour samples had an average contamination level of 4.9 ppb, compared to 9.9 ppb for posho mill samples (p < 0.001). Contamination levels were lowest among national brands (2.8 ppb) compared to regional brands (5.6 ppb). This difference is statistically significant (p = 0.001). There is notable heterogeneity across brands of packaged flour with mean contamination levels varying between 1.2 ppb for the least contaminated brand to 32.0 ppb for the brand with the highest aflatoxin level. Flour milled from home-grown grains was less contaminated (7.4 ppb) than flour milled from purchased grains (10.3 ppb), but the number of home-grown samples was limited, and this difference is not statistically significant (p = 0.218).

**Fig 5:**
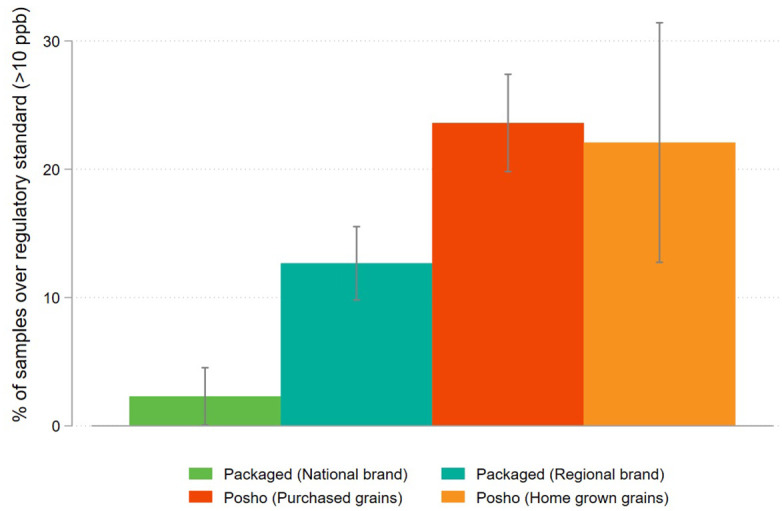
Share of maize flour samples exceeding the national regulatory standard for aflatoxin (>10ppb), disaggregated by flour source.

Differences in contamination above the regulatory limit of 10ppb are also apparent. For branded samples, 10% were above this limit (2% of national brand samples, 13% of regional brand samples), whereas approximately one in four posho mill flour samples were above the limit (Fig 5). The proportion of posho milled from home grown grains over the limit was approximately the same as for posho milled from purchased grains.

**Fig 6:**
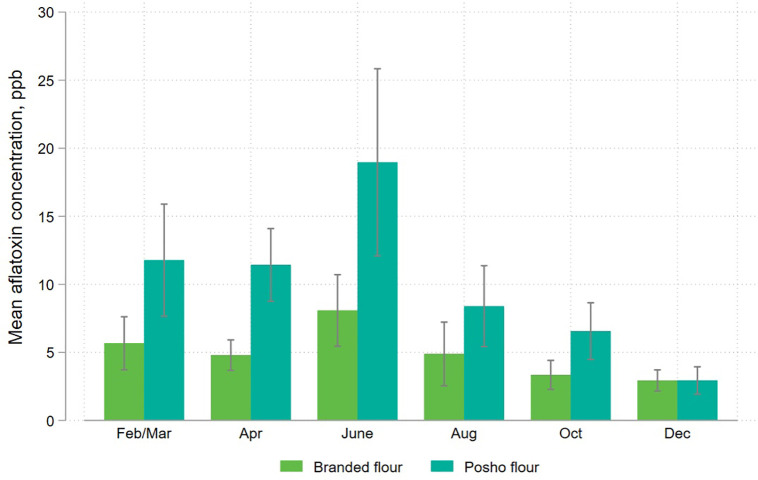
Mean aflatoxin levels in packaged and informally processed maize flour, disaggregated by sampling round.

**Fig 7:**
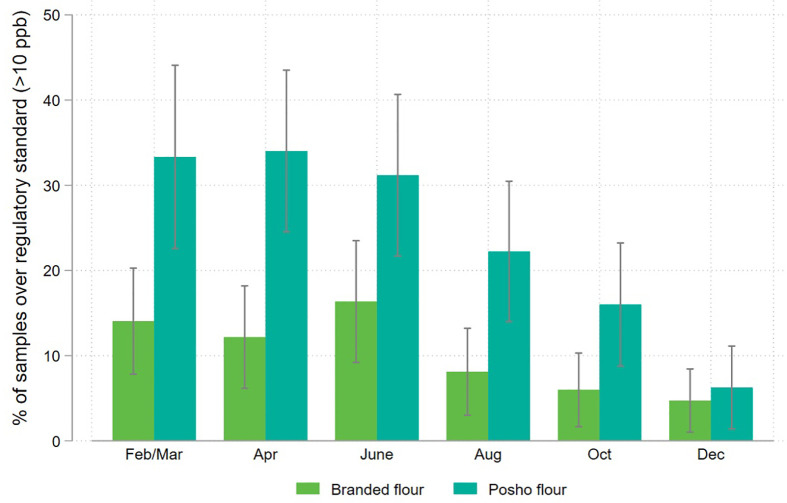
Share of packaged and informally processed maize flour samples exceeding the national regulatory standard for aflatoxin (>10ppb), disaggregated by sampling round.

Figs 6 and 7 disaggregate these results by sampling round and location, pooling national and regional brands, and the grain source of posho samples.

The pattern of higher contamination in posho versus packaged flour is consistent throughout the year. Contamination is highest for both branded and posho flour in June, and dips in October through December, as maize from the Rift Valley, a productive region with relatively low aflatoxin risk, comes onto the market.

**Fig 8:**
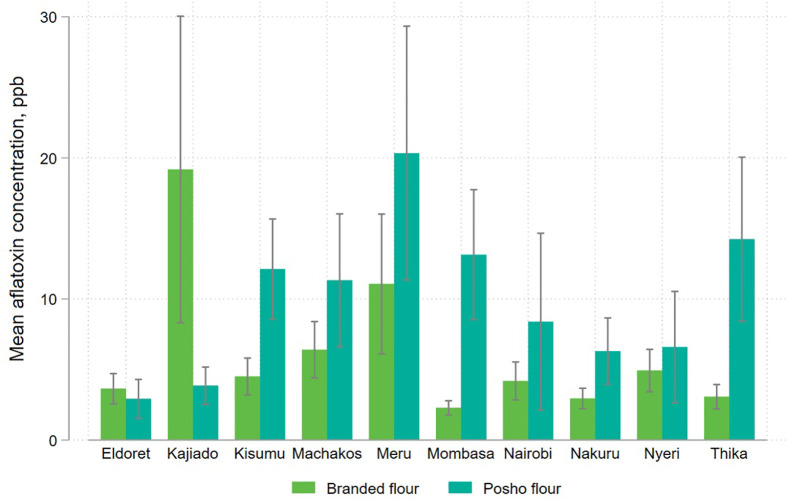
Mean aflatoxin levels in packaged and informally processed maize flour, disaggregated by mill location.

**Fig 9:**
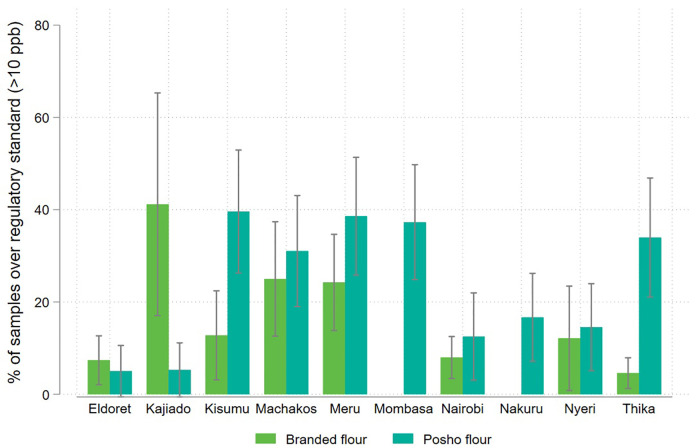
Share of packaged and informally processed maize flour samples exceeding the national regulatory standard for aflatoxin (>10ppb), disaggregated by mill location.

We observe a high degree variation by milling location, with the highest levels of contamination observed in the eastern sample collection sites of Kajiado, Meru, and Thika. In sites where contamination levels differed significantly across flour types, posho flour was almost always more contaminated than formally milled flour. The exception is Kajiado, where more than 41% of formally milled flour samples (all of same brand) tested above the regulatory threshold. The regulatory standard of 10ppb falls within the 95% confidence interval of mean contamination in branded flour in Kajiado and Meru, and for posho flour in Kisumu, Machakos, Meru, Mombasa, Nairobi, Nyeri and Thika, indicating a substantial risk of aflatoxin contamination in maize produced in these regions (Figs 8 and 9).

**Fig 10:**
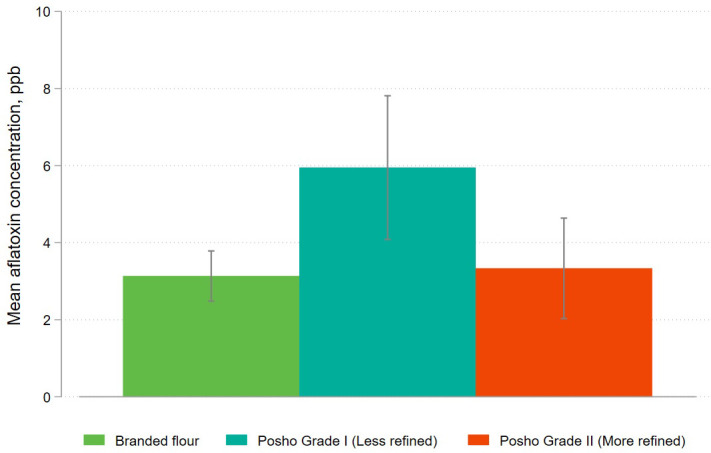
Mean aflatoxin levels for packaged and informally processed maize flour, disaggregated by level of processing (Sampling Rounds 5 & 6).

**Fig 11:**
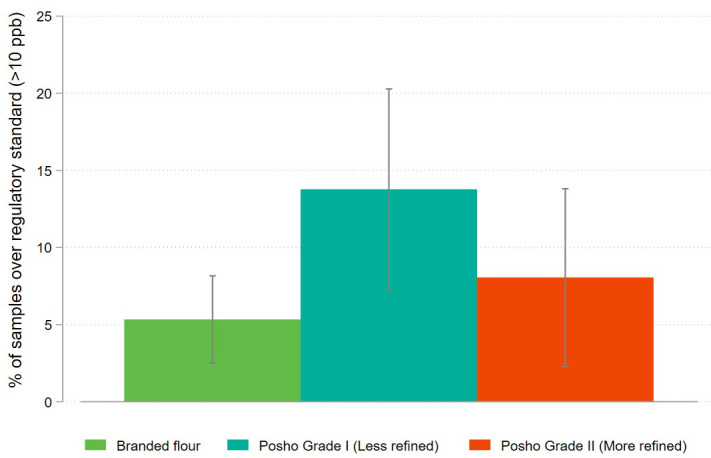
Share of packaged and informally processed maize flour samples exceeding the national regulatory standard for aflatoxin (>10ppb), disaggregated by level of processing (Sampling Rounds 5 & 6).

Lastly, we consider how differential removal of the germ and bran during the milling process may explain the difference in aflatoxin contamination observed between posho and packaged maize flour. Packaged flour is generally more refined – meaning that more of the germ and bran have been removed – than posho flour. There is some variation in the degree of refining performed by posho mills, with many mills differentiating between grade 1 (less refined, cheaper flour) and grade 2 (more refined, higher priced flour). While processes are not uniform across posho mills, a majority of posho mills in our sample report selling multiple grades of flour. To test differences across grades, for the final two rounds of data collection, laboratory technicians assigned a grade based on visual inspection to each posho sample (Figs 10 and 11).

The results for packaged flour and the more refined grade of posho are very similar, with an average contamination level of 3.1 ppb and 3.3 ppb respectively, and the difference between the two is not statistically significant (p = 0.815). The difference in aflatoxin contamination levels across posho versus packaged flour for this sub-sample is therefore driven primarily by higher contamination in less refined posho flour, which exhibited an average contamination rate of 5.9 ppb (p < 0.001). The share of samples above the regulatory limit is 5.3% for branded flour, 8.0% for more refined posho flour, and 13.8% for less refined posho flour. The difference in proportions above the limit is not statistically significant between branded and more refined posho flour (p = 0.364) but is significant when comparing branded and less refined posho flour (p = 0.009).

These results suggest that that the lower level of aflatoxin contamination found in packaged maize flour is due to the fact that the non-starch components of the grain are more fully removed during the formal milling process, relative to typical posho milling. Aflatoxin tends to be most concentrated in the germ and bran of the maize kernel, the components sifted out the refining process [15]. These are also the most nutritionally dense components of the whole grain, implying a potential tradeoff between nutrition and food safety. This tradeoff is mitigated by mandatory fortification of formally processed maize flour in Kenya. Compliance with micronutrient fortification requirements is far from perfect in Kenya, with the proportion of maize samples compliant with fortification standards ranging from 23% to 49% as of 2018 [16]. Even so, at these levels of compliance, the average levels of vitamin A, zinc, and iron would be higher in branded maize than in whole grains (Table 3).

**Table 3:** Relative levels of key micronutrients in whole maize vs. fortified flour (mg/kg).

	(1) Fortification standard^1^	(2) Proportion compliant^1^	(3) Mean level in branded maize = (1) x (2)	(4) Whole grain^3^
Vitamin A	0.5	0.23	0.1	0
Zinc	33	0.34	11.2	2.21
Iron	21	0.49	10.3	2.71

*Notes*: Fortification standards and compliance data are taken from the Kenyan Ministry of Health. These are multiplied to compute the mean level in branded maize. Levels in whole grain maize are taken from Gwirtz and Garcia-Casal [17].

## Discussion

This study presents aflatoxin contamination results from the most temporally and spatially comprehensive sample to date of retail maize products in Kenya. Previous research characterizing the aflatoxin contamination of maize in Kenya has typically been specific to a particular region and timepoint [6–10]. Further, aside from one study conducted in a single municipality with a limited sample sizes [19], no prior work has compared contamination levels of sifted maize flour processed in large-scale mills against that of whole-grain maize processed in small-scale hammer mills.

Maize is transported across Kenya throughout the year from maize surplus to maize deficit regions. This variation is driven by differences in the timing of the growing season, so the composition of available maize that is locally produced (rather than produced in a different region) varies over the course of a year. Capturing variation in aflatoxin contamination at the retail level across time for a range of locations is thus informative for capturing consumer exposure to this hazard. Since a miller in a particular region may source maize locally during some months and use maize grown elsewhere at other times and since we lack information on the source of maize, our methodology is expected to understate the extent of geographical variation in aflatoxin contamination at the producer level. However, the variation we observe, especially if combined with information on maize sources obtained from traders or millers, can still help identify areas of higher production risk to which additional on-farm sampling could be targeted.

The results of this study mirror the previously observed spatial pattern of population aflatoxin exposure in Kenya, with the highest level of contamination in maize milled in the eastern part of the country, and lower levels in the high-productivity maize basket of Rift Valley [18]. The finding that contamination in whole meal posho flour is higher than in formally milled, sifted flour is congruent with previous evidence based on samples collected in Meru town [19]. Our analysis of posho flour by grade suggests that the difference in contamination between branded, sifted maize flour, and informally milled whole meal maize flour arises through the removal of germ and bran during the sifting process of packaged maize flour production.

Monitoring aflatoxin contamination levels across locations in Kenya throughout the year has the potential to better target public and private resources for aflatoxin control by directing resources to the parts of the country where, and seasons when, risk is highest. Further, information about the relative risk of aflatoxin contamination in alternative maize products could help consumers make safer food choices. While formally processed maize flour is becoming increasingly important in Kenya, informally milled posho flour remains the primary staple, especially in rural areas [20]. Previous research has shown that consumers respond to food safety information by switching to lower-risk substitutes [19]. Informing consumers that national brands are relatively safe, and that posho flour is relatively risky, could potentially expand the market share of the larger national brands and sifted maize flour in general, and in this way reduce population-level aflatoxin exposure. Currently, consumers are given only partial information, due to the regulatory focus on formally registered, branded products. Recalls of maize flour by national and county regulatory authorities in Kenya are increasingly common [21–23]. In the absence of comprehensive information on the relative risk of different products, such recalls could potentially lead consumers to switch to riskier posho flour. Providing more complete information on the relative risks across maize products could avoid this unintended consequence.

A similar surveillance strategy could be applied to other food types and hazards. For example, microbiological contamination in high-risk foods such as dairy or horticultural products could be systematically monitored by region through the purchase of formal sector packaged, formal sector unpackaged (supermarket, milk dispenser) and informal unpackaged samples.
